# Efficacy of Docosahexaenoic Acid for the Prevention of Necrotizing Enterocolitis in Preterm Infants: A Randomized Clinical Trial

**DOI:** 10.3390/nu13020648

**Published:** 2021-02-17

**Authors:** Mariela Bernabe-García, Philip C. Calder, Raúl Villegas-Silva, Maricela Rodríguez-Cruz, Luis Chávez-Sánchez, Leonardo Cruz-Reynoso, Leovigildo Mateos-Sánchez, Gabriel Lara-Flores, Augusto R. Aguilera-Joaquín, Luisa Sánchez-García

**Affiliations:** 1 Unidad de Investigación Médica en Nutrición, UMAE Hospital de Pediatría, CMN Siglo XXI, Instituto Mexicano del Seguro Social, México City 06720, Mexico; marielabernabe1@gmail.com (M.B.-G.); maricela.rodriguez.cruz@gmail.com (M.R.-C.); 2 School of Human Development and Health, Faculty of Medicine, University of Southampton, Southampton SO16 6YD, UK; 3 Institute for Life Sciences, University of Southampton, Southampton SO17 1BJ, UK; 4 NIHR Southampton Biomedical Research Centre, University Hospital Southampton NHS Foundation Trust and University of Southampton, Southampton SO16 6YD, UK; 5 Neonatología, Hospital Infantil de México Federico Gómez, México City 06720, Mexico; raul.villegassilva@gmail.com; 6 Unidad de Investigación Médica en Inmunología, UMAE Hospital de Pediatría, CMN Siglo XXI, Instituto Mexicano del Seguro Social, México City 06720, Mexico; luischz@yahoo.com; 7 Unidad de Cuidados Intensivos Neonatales, UMAE Hospital de Gineco-Obstetricia No.3, CMN La Raza, Instituto Mexicano del Seguro Social, México City 02990, Mexico; drleonardocruz@yahoo.com.mx (L.C.-R.); draguilera357@gmail.com (A.R.A.-J.); lsanchezg60@hotmail.com (L.S.-G.); 8 Unidad de Cuidados Intensivos Neonatales, UMAE Hospital de Gineco-Obstetricia N° 4 “Luis Castelazo Ayala”, Instituto Mexicano del Seguro Social, México City 01090, Mexico; lmateos95@yahoo.com.mx (L.M.-S.); drdebebes@gmail.com (G.L.-F.)

**Keywords:** very low birth weight, infant, prematurity, necrotizing enterocolitis, inflammation, DHA, omega-3, n-3 fatty acids, neonatal intensive care unit, hospital stay

## Abstract

Necrotizing enterocolitis (NEC) is an inflammatory bowel disease and a leading cause of morbidity and mortality in preterm infants. In this study, a randomized double-blind parallel-group (1:1) trial was carried out in two neonatal intensive care units of two tertiary hospitals. Two hundred and twenty-five preterm newborns with an expected functional gastrointestinal tract were recruited and received an enteral dose of 75 mg of docosahexaenoic acid (DHA)/kg body weight or high-oleic sunflower oil daily for 14 days from the first enteral feed after birth. Confirmed NEC was evaluated with Bell’s scale from stage ≥ IIa. Two hundred and fourteen randomized infants were analyzed in terms of the intent-to-treat (DHA-group: *n* = 105; control-group: *n* = 109); data for two hundred infants were analysed per protocol. Confirmed NEC was lower in infants from the DHA-group compared with the control-group (0/100 vs. 7/100; *p* = 0.007), with RR = 0.93 (95% CI 0.881 to 0.981), risk difference = −7%, (95% CI −12.00 to −1.99), and number needed-to-treat = 15 (95% CI 8.3 to 50). Intent-to-treat analysis showed a lower level of treatment failure in the DHA-group compared with the control-group (6/105 (6%) vs. 16/109 (15%); *p* = 0.03, RR = 0.905, (95% CI 0.826 to 0.991)). The results after multivariate-regression analysis remained significant. Adverse events (apart from the incidence of NEC) were not different between groups. A daily dose of DHA for 14 days starting with the first enteral feed may prevent NEC in preterm infants.

## 1. Introduction

Necrotizing enterocolitis (NEC) is a multifactorial inflammatory bowel disease. This condition starts with an unbalanced pro-inflammatory response that rapidly evolves without warning into a necrotic bowel and/or death. Most cases occur in preterm infants (birth weight < 1500 g or < 32 weeks of gestational age) [1,2], and NEC remains a leading cause of morbidity and mortality in neonatal intensive care units (NICUs) worldwide [3]. Moreover, survivors of NEC have long-term complications and high medical costs [4].

The pathophysiology of NEC involves bowel and immune immaturity, including scarce mucus, low secretory immunoglobulin A, poor closure between enterocytes along with an altered intestinal bacterial diversity (dysbiosis) related to antibiotic administration, and the type of feeding, among other factors, especially in formula-fed infants [5,6]. Once developed, NEC is managed through support measures [1]. Therefore, strategies to prevent NEC are needed. 

Docosahexaenoic acid (DHA), which is an n-3 long-chain polyunsaturated fatty acid (LC-PUFA), is accreted during the last trimester of gestation [7]. This accretion is cut-off by a preterm birth, so preterm infants can become deficient in DHA after birth, even if they receive LC-PUFA supplemented formula [8]. DHA is found in human breast milk, which reduces the risk of NEC [7]. A global survey of human breast milk fatty acids using data from 65 studies involving 2474 women reported a mean (SD) DHA content of 0.32 (0.22) percent of total fatty acids, with a range of 0.06 to 1.4% of total fatty acids [9]. The DHA content of breast milk changes with the duration of lactation and is highest in colostrum [10]. Some, but not all, infant formulas contain DHA, typically including about 0.3% of total fatty acids [11]. DHA exhibits actions that can modulate exacerbated inflammatory responses in several neonatal morbidities, including NEC [12]. 

Therefore, the aim of this study was to evaluate the efficacy of the enteral administration of DHA to prevent NEC in preterm infants; considering that preterm formula may be an inflammatory stimulus, the intervention started with the first enteral feed after birth.

## 2. Materials and Methods 

### 2.1. Study Design and Participants

A randomized double-blind parallel-group clinical trial was conducted in preterm newborn infants with birthweight ≤ 1500 g, but ≥ 1000 g, with an expected functional gastrointestinal tract; infants were recruited between October 2012 and October 2017 from two hospitals affiliated with the Instituto Mexicano del Seguro Social (IMSS) in México City. Infants with congenital malformations, a need for major surgery, or periventricular/intraventricular hemorrhage grade ≥ II were excluded. At recruitment, none of the mothers were taking n-3 fatty acid supplements or had taken them during pregnancy. 

This research was carried out in accordance with The Code of Ethics of the World Medical Association [13] and was approved by the Ethics Committee, National Committee of Scientific Research from IMSS (institutional code CNIC-2012-785-007). The trial was registered at clinicaltrials.gov (NCT01745510) prior to the enrolment of the first participant. Written informed consent was obtained from both parents/guardians (overseen by two witnesses) prior to infant randomization, conforming to the Regulation of the General Law of Health, in matters of Research for Health in Mexico [14]. 

### 2.2. Randomization and Intervention

Randomization was performed using the Random Allocation Software version 1 for parallel groups, with a 1:1 intervention ratio and block sizes of 10 patients per birthweight (1000–1250 g and from 1251–1500 g) [15]; each hospital had its own independent randomization assignment. The intervention groups received a code (A or B) and were packed into opaque envelopes by a researcher who did not participate in the fieldwork; all other clinical and research staff were blind to allocation.

Preterm infants received a daily dose of 75 mg of DHA/kg of body weight (DHA-group) from a DHA-rich algal oil diluted in high-oleic sunflower oil as a vehicle (Neuromins^®^ for Kids life’s DHA^®^; DSM Nutritional Products Inc., Parsippany, NJ, USA) or sham oil (control group; high-oleic sunflower oil; PROGELA SA, México City, Mexico) for 14 days, similar in colour and consistency. The fatty acid composition of the oils is shown in Table 1. 

Every 3 days, research staff updated the infant’s weight to modify the dosage. Research staff directly observed administration of the oils, which were given from the first feed after birth, being flushed through an orogastric tube before the milk and/or enteral formula. The DHA dose mimics the high physiological supply of DHA from human milk [9] and has been well-tolerated in previous studies in neonates [16,17]. DHA administration was suspended if any persisting bleeding was identified, if the platelet count was < 80,000 mm^3^, or if the infant was fasting due to acute illness. 

### 2.3. Main Outcome Measure: Confirmed Necrotizing Enterocolitis 

Confirmed NEC was determined prospectively using Bell’s scale modified by Walsh [18]. Briefly, stages Ia to Ib are suspected NEC, stages IIa to IIb are confirmed NEC, and severe/advanced NEC is from stage IIIa with an intact bowel and stage IIIb with a perforated bowel. The attendant neonatologist identified the concordance of the X rays with systemic and intestinal signs. Then, a second neonatologist confirmed or discarded the diagnosis. In the case of non-concordance, a third neonatologist decided the diagnosis. Confirmed NEC was considered from stage IIa or greater [18].

### 2.4. Adverse Events

Adverse events included a platelet count < 80,000 mm^3^ (collected from routine biochemical analyses), bleeding events such as periventricular/intraventricular hemorrhage grade ≥ II, pulmonary and gastric bleeding, and death. 

### 2.5. Clinical Course and Management

At recruitment, infant sex, gestational age, presence of severe asphyxia at birth, being small for the gestational age, being a twin or singleton, and the severity of disease measured with the Clinical Risk Index for Babies (CRIB) Score [19] were recorded. The clinical course, such as the presence of patent ductus arteriosus, respiratory distress syndrome, suspected sepsis, and apnea events, as well as medical management, such as antibiotics, ibuprofen, omeprazole, red blood cell (RBC) transfusions, and the duration of low oxygen saturation (SpO_2_ < 85%), were recorded. 

Details on nutritional support were collected until hospital discharge. Patients who required total/partial parenteral nutrition (TPN) received a 20% soybean oil-based lipid emulsion. Intake of the mother’s own milk was recorded. Data are presented for the first 2 weeks of the intervention and total hospital stay. Hospital discharge happened when the infant was able to maintain a stable body temperature and had reached a body weight of at least 1.8 kg.

### 2.6. Fatty Acid Profile in Erythrocytes and Human Milk 

The fatty acid profile of infant erythrocytes was measured. To avoid additional punctures for research purposes, an additional blood sample was collected in ethylenediaminetetraacetic acid tubes when consultants ordered blood for clinical tests. The method employed to measure the erythrocyte fatty acid profile by gas chromatography has been reported elsewhere [17]. Briefly, after total lipid extraction from erythrocytes by the modified Folch method, fatty acid methyl esters (FAMEs) were produced and separated by gas chromatography (Agilent 7820A, Agilent Technologies, Santa Clara, CA, USA). The identification of FAMEs was conducted according to the retention time from specific FAME standards (Poly Sciences, Niles, IL, USA) and heptadecanoic acid was employed as an internal standard [20]. The fatty acid profile of human milk samples was determined using the same methods. Fatty acids are reported as the weight percentage of total fatty acids (%wt of total fatty acids). The fatty acid profile of the enteral formula was estimated from the composition reported by the manufacturers.

### 2.7. Sample Size and Statistical Analysis

The sample size was estimated with a two-sided α = 0.05 and a power = 80%, where P1 for control = 15% and P2 for DHA intervention = 4%. A sample size of 111 patients/group was obtained. However, a bivariate interim analysis performed after 5 years identified significant differences between groups and recruitment was stopped.

IBM SPSS software version 21 (IBM Corp., Armonk, NY, USA) was used for data analysis. The data distribution was inspected. Data are presented as the median and interquartile range (quartile 25, quartile 75) or (minimum, maximum) for variables with scarce patients. DHA and control groups were compared with the Mann–Whitney-U test, Chi-square test, and relative risk (RR) with a 95% confidence interval (CI). However, as both groups had similar covariates, the infants in the control-group who developed NEC were also compared as an independent group with the DHA-group and the remainder of the infants in the control-group without NEC, using Fisher´s Exact, Kruskal–Wallis, and Mann–Whitney-U tests. Multivariate analysis with decision trees using Chi-square automatic interaction detection (CHAID) regression was applied to identify the predictors of confirmed NEC. In all cases, a *p* value < 0.05 was considered to be statistically significant. Those preterm infants who received at least one dose of DHA or sham were included in the intent-to-treat analysis using the Chi- square test and RR (95% CI). 

## 3. Results

**Figure 1:**
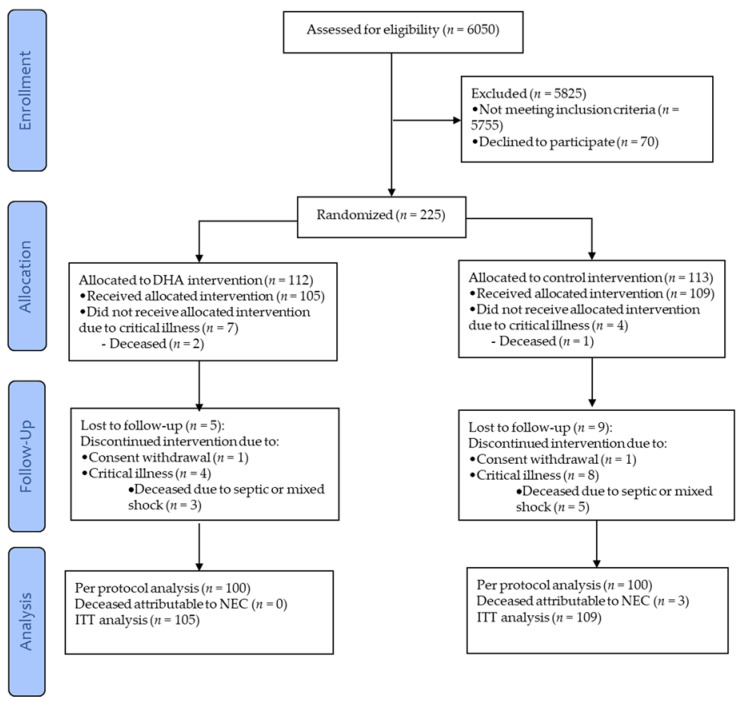
CONSORT diagram depicting the flow of the infants through the study. DHA, docosahexaenoic acid; NEC, necrotizing enterocolitis; and ITT, intent-to-treat.

From the two hospitals, 225 preterm infants were recruited and randomized; 214 infants received at least one dose of DHA or sham and from them, 100 infants per group were analysed per protocol (Figure 1). Of these 200 infants, 179 were recruited at one hospital (*n* = 90 in the control-group, *n* = 89 in the DHA-group) and 21 at the other (*n* = 10 in the control-group, *n* = 11 in DHA-group). Baseline characteristics of the infants were comparable between DHA and control groups (Table 2).

### 3.1. Confirmed Necrotizing Enterocolitis

Seven infants developed NEC and all were in the control-group. Therefore, infants with NEC were separated from the rest of the infants in the control-group, but the characteristics remained similar (Table 3). All infants who developed NEC had sepsis prior to developing NEC and all were from one of the two hospitals; this was the hospital that recruited the higher number of infants.

Confirmed NEC was lower in infants from the DHA-group compared with the control-group (0/100 vs. 7/100, *p* = 0.007) with a RR of 0.93 (95% CI 0.881 to 0.981, *p* = 0.008); there was a risk difference of −7% (95% CI −12.00 to −1.99), and a number needed to treat of 15 (95% CI 8.3 to 50). Among the NEC cases, four were female and three were male. The stages of NEC were two cases of IIa, three cases of IIb, one case of IIIa, and one case of IIIb.

The median (minimum, maximum) postnatal age at diagnosis of confirmed NEC was 26 (6, 29) days. The post-enteral feeding time to diagnosis of confirmed NEC was 4 (0, 23) days. 

The analysis according to intent-to-treat (ITT) showed a lower level of treatment failure in the DHA-group compared with the control-group (6/105 (6%) vs. 16/109 (15%); *p* = 0.031) with an RR of 0.905 (95% CI 0.826 to 0.991). 

**Figure 2:**
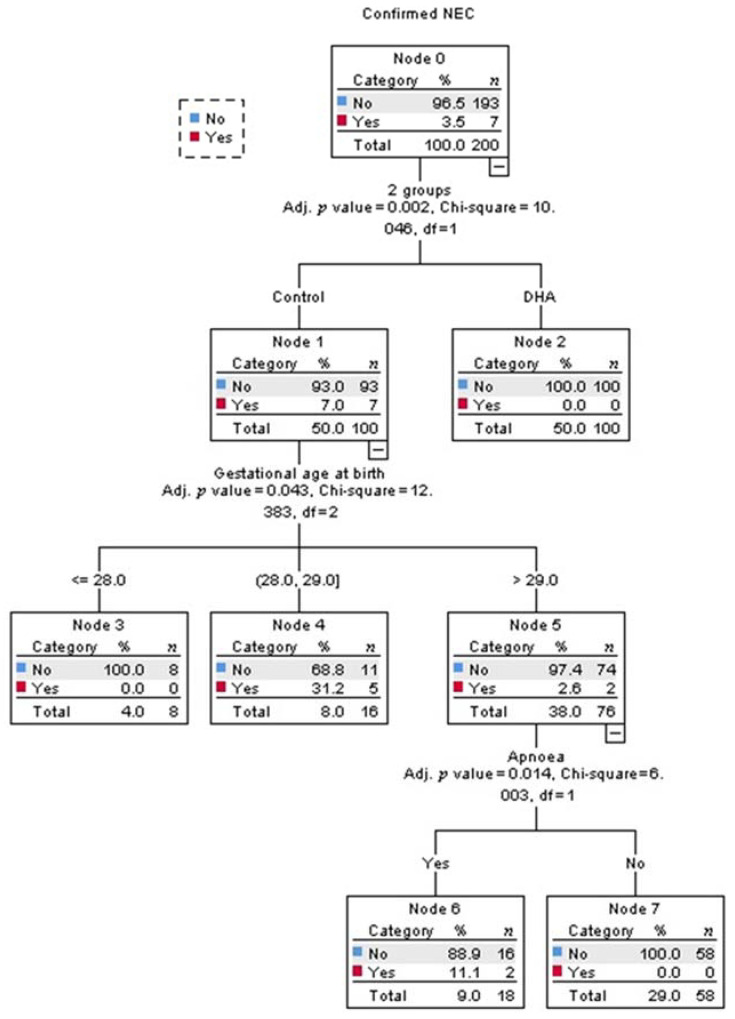
Decision tree obtained from Chi-square automatic interaction detection multiple regression analysis for the prediction of confirmed necrotizing enterocolitis (NEC). The order of the variables, from top to bottom, shows their ranking for the prediction of confirmed NEC; the first was the intervention, the second was gestational age, and the last was presenting apnea.

**Figure 3:**
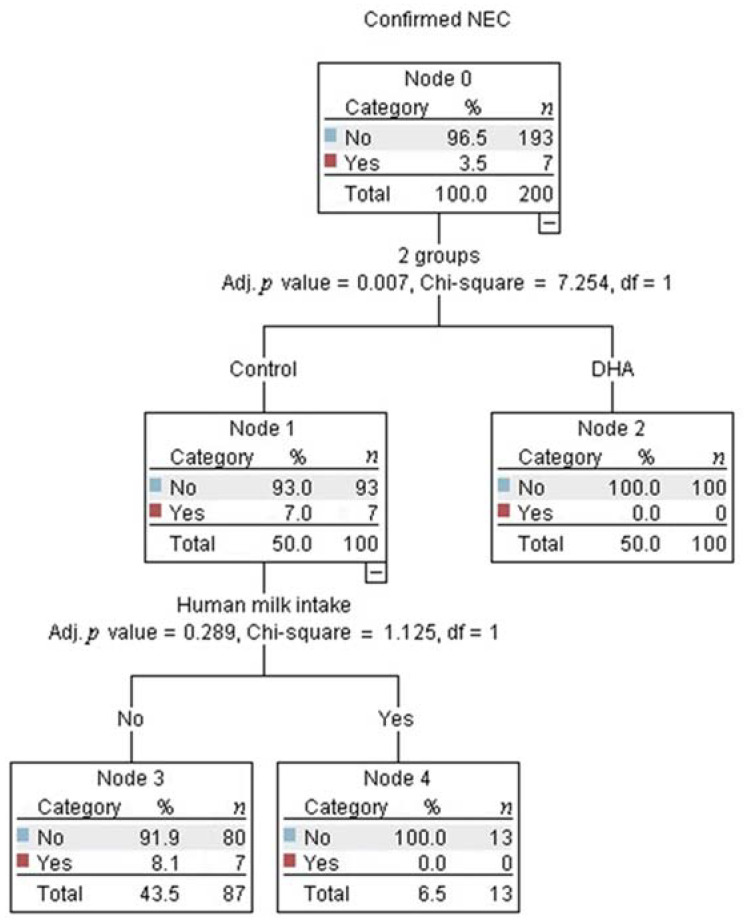
The decision tree obtained from the Chi-square automatic interaction detection multiple regression analysis showed that no infants in the control group who received any volume of human milk developed necrotizing enterocolitis (NEC).

Multivariate analysis identified that being in the control-group was the strongest predictor for developing NEC (adjusted *p* = 0.002, Figure 2). Among infants in the control-group, the best predictor for developing NEC was the gestational age (adjusted *p* = 0.043, Figure 2). The probability of developing NEC was higher in infants older than 29 weeks of gestational age at birth who presented with apnea (adjusted *p* = 0.014, Figure 2). The intake of any volume of the own mother’s milk was a non-statistically significant, but clinically protective predictor of NEC in the control-group (Figure 3).

### 3.2. Adverse Events

The median (minimum, maximum) of platelet counts was not different between the DHA and control groups at the baseline and after the intervention (196,000 mm^3^ (107,000, 464,000) vs. 182,000 (103,000, 473,000), *p* = 0.647 and 345,000 mm^3^ (120,000, 598,000) vs. 347,000 (131,000, 776,000), *p* = 0.884, respectively). No infant showed a platelet count <80,000 mm^3^.

The per protocol analysis showed no difference in death between the DHA and control groups (1/100 compared to 5/100, one-sided hypothesis *p* = 0.106), with an RR of 0.960 for the DHA-group (95% CI 0.914 to 1.008). Of the five deaths in the control-group, three were attributable to NEC; the death in the DHA-group was not attributable to NEC. Likewise, in the intent-to-treat analysis, mortality was not different between the DHA-group and control-group (3/105 vs. 8/109, *p* = 0.119) with an RR = 0.954 for infants in the DHA-group (95% CI 0.896 to 1.015).

During the total hospital stay, there was no difference between the DHA and control groups in the presence of at least one of the following bleeding entities: periventricular/intraventricular hemorrhage grade ≥ II and upper gastrointestinal tract and/or pulmonary bleeding (68/100 compared with 73/100, *p* = 0.438). The median of the bleeding events was similar (1 (1, 2) vs. 1 (1, 2), *p* = 0.814). Periventricular/intraventricular hemorrhage grade ≥ II was the most common type of bleeding (57% in the DHA-group vs. 54% in the control-group, *p* = 0.669). 

### 3.3. Clinical Course and Management

Covariates from the clinical course and management were similar and there was no collinearity among them. Therefore, data were stratified by the two treatments and the infants with confirmed NEC were employed as an independent group (Table 3).

Regarding nutritional support, total parenteral nutrition did not contain eicosapentaenoic acid, arachidonic acid (AA), or DHA. The volume of fluids, osmolarity (239 mOsm/L), and enteral formula were not different among groups. The enteral formula for preterm infants contained 19.4 mg of both DHA and AA per 100 kcal; the cumulative intake of DHA and AA from the formula was not different (Table 4). The cumulative intake of DHA and AA estimated from human milk, in those infants receiving human milk during the two weeks of intervention, displayed a median of 0.31 (0.12 to 1.13) mg/kg/day and 0.84 (0.35 to 2.52) mg/kg/day in the DHA-group, while the control-group received 0.25 (0.12 to 0.38) mg/kg/day and 0.35 (0.18 to 0.63) mg/kg/day, respectively. Infants in the DHA-group received an additional 75 mg DHA/kg body weight/day. The infants who developed NEC showed a non-statistical trend of a higher postnatal age when starting enteral feeding and receiving lower advances in enteral feeding (maximum increases do not exceed 25 mL/kg/day), resulting in a statistical trend of lower nutritional support (Table 4). No infant who developed NEC received breastmilk (Table 4), and as consequence, they did not receive fatty acids from human milk (Table 5). Other aspects of nutritional support were not different among groups. 

### 3.4. Fatty Acid Profile in Erythrocytes and Human Milk

The fatty acid profile of erythrocytes collected at the baseline was similar between groups, except that alpha-linolenic acid was higher in the DHA-group (Table 5). The human milk received by the infants in the DHA-group contained less eicosapentaenoic acid and DHA than the human milk received by the infants in the control-group; the remaining fatty acids were similar for the human milk of both groups, including AA (Table 5).

## 4. Discussion

This trial demonstrates that the enteral administration of 75 mg/kg/day of DHA starting at the first enteral feed prevents NEC in preterm infants. The efficacy of DHA remained significant in both intent-to-treat and multivariate analysis. To the best of our knowledge, this is the first trial to evaluate the efficacy of isolated enteral DHA administration on confirmed NEC.

Our findings are consistent with those of Lu et al., who found a reduced NEC incidence (25%) in neonatal rats that received a formula with DHA (0.5% of fatty acids) compared with the incidence of 35% in those with AA (0.7%) plus DHA (0.5%) and 50% in the control-group [21]. Although that study did not specify whether neonatal rats were preterm, a separate study found similar results in preterm rats: 26% receiving DHA (0.5%) developed NEC, compared with 35% in a group with AA (0.7%) plus DHA (0.5%) and 50% in controls [22]. Those studies suggest a more protective effect of DHA in the absence of AA. Although AA is necessary for growth and cognitive development [11], it is also considered to have some pro-inflammatory properties [12].

Interestingly, an in vitro study using epithelial cells from a resected small intestine from a neonate with NEC demonstrated that treatment with DHA significantly decreased the interleukin (IL)-1beta-induced production of pro-inflammatory cytokines IL-8 and IL-6 compared with controls; AA did not exert an effect on those cells [23]. 

Our findings are, to some extent, consistent with the results of Carlson et al., who reported a reduced incidence of NEC in infants receiving a formula with AA (0.41%) plus DHA (0.13%) compared with a control formula, for which the NEC incidence was 2.9% and 17.6%, respectively [24]. However, that study showed a high incidence of NEC. In another study, Innis et al. found 0, 2, and 1 cases of suspected/confirmed NEC in preterm infants receiving AA (0.60%) plus DHA (0.33%), DHA (1%), or a control formula, respectively [25]. In that study, NEC was sometimes suspected rather than confirmed and the incidence was low. 

A meta-analysis [26] and a single study in preterm infants [27] reported no effect of formulas supplemented with AA plus DHA for preventing NEC. The authors of the meta-analysis reported that studies had small sample sizes and a high risk of bias. A large study that administered a dosage of DHA similar to in utero accretion (60 mg/kg/day) did not find differences in bronchopulmonary dysplasia (primary outcome) or NEC [28]. It is noteworthy that these studies estimated their power based on primary outcomes other than NEC. Therefore, they may be under-powered for detecting an effect on NEC or they may inaccurately evaluate NEC (some studies do not report how NEC was evaluated). The present study had NEC as its primary outcome, selected a population at risk of NEC, and was powered accordingly. It is possible that the preterm infants from our study may be better responders to DHA compared with infants in other studies. This is speculated because a low maternal intake of DHA results in a poor neonatal status: A study from central Mexico showed a low maternal intake of DHA (median 0.11 mg/day) [29]. In the current study, infant erythrocyte DHA averaged ~3% of total fatty acids. This is lower than reported in many other studies. For example, in a recent US study, DHA in erythrocytes from 100 one-month-old infants ranged from 3.96% to 7.75% [30]. 

Preterm infants have enterocytes prone to inflammatory responses. This has been explained to be due to exaggerated toll like receptor (TLR) 4 expression and inflammatory signaling and can upregulate platelet-activating factor (PAF) production, which plays an important role in the pathogenesis of NEC. Preterm infants also exhibit under-expression of the inhibitory sub-unit (IƙB) of the nuclear transcription factor (NF) ƙB, which results in the easier synthesis of pro-inflammatory cytokines, chemokines, and other inflammatory mediators [2,31]. Some studies have shown that pro-inflammatory cytokines can weaken intestinal barrier function, escalating inflammation, injury, and gut damage and enabling bacteria from the bowel to enter the circulation [32,33]. Moreover, monocytes from preterm infants produce lower levels of IL-10 [34], which is an anti-inflammatory cytokine critical for intestinal homeostasis [5], increasing the risk of exaggerated bowel inflammation. 

There is much evidence that DHA reduces inflammation, preventing gut disruption due to several mechanisms, such as decreased NFƙB activation; decreased pro-inflammatory cytokine and PAF synthesis; and the increased production of pro-resolving mediators, such as resolvins, protectins, and maresins, which in turn reduce cytokine production and inflammatory cell recruitment [12,35].

The dose of DHA used in the current study (75 mg/kg/day) was used peri-operatively in term neonates who underwent cardiovascular surgery [17]. In that study, circulating monocytes showed an early increase of anti-inflammatory cytokine expression (IL-1 receptor antagonist and IL-10), along with a non-significant increase of pro-inflammatory cytokine expression (IL-1β, TNF-α, and IL-6) [17]. Therefore, this DHA dosage seems relevant for modifying inflammatory processes and switching to a less inflammatory state. It is biologically plausible that the administration of DHA in anticipation of an inflammatory challenge, as in the current study, represents a narrow window of opportunity for a protective anti-inflammatory strategy. The approach is feasible and inexpensive; the dose of DHA-containing oil can be adjusted and flushed before the milk or enteral formula with a syringe coupled to a feeding tube. 

Regarding covariates of the clinical course and medical management, in the current study, the second predictor of NEC (after treatment allocation) in multivariate analysis was gestational age, consistent with the developmental window for the susceptibility of NEC onset [6,36]. The third predictor was apnea events, which result in intestinal hypoxia/ischemia. Nonetheless, these were only significant predictors in the control-group; there was no predictor of NEC in the DHA-group, since NEC did not occur in that group (Figure 2). All other potential confounders were not significant and therefore, the regression did not select them. 

Human milk from mothers with infants in the control-group had higher DHA and eicosapentaenoic acid levels (Table 5), but the estimated cumulative intake of DHA, although negligible, tended to be higher in the DHA-group than in the control-group. This is explained by the fact that the average volume of human milk received in the DHA-group tended to be higher than in the control-group. Additionally, the human milk intake per week did not increase steadily due to critical illness in those infants, but was different among groups (Table 4). Therefore, to evaluate the effect of human milk on confirmed NEC, it was intentionally added along with the treatment allocation in the regression model. Although the effect of human milk on confirmed NEC was not statistically significant, this analysis confirmed a clinically significant effect because it separated those infants from the control-group receiving any volume of human milk and no development of NEC (right square, Figure 3). This analysis was not possible in the DHA group because this group had no NEC events. It is well-known that bioactive factors contained in human milk protect against the onset of NEC [37], giving an absolute risk reduction of 4% for any stage of NEC for preterm infants [38]. Interestingly, in the present study, the absolute risk reduction with DHA was 7%. Therefore, this intervention should be considered as a strategy for NEC prevention. In this study, preterm birth was often due to maternal critical illness. Therefore, only 31/200 infants received any volume of human milk, 15/200 received it for at least 2 weeks, and no infant was exclusively-fed with human milk. 

A previous review identified that antenatal steroids, such as ibuprofen/indomethacin, for the treatment of patent ductus arteriosus were protective factors, while lower oxygen saturations (85–89%) increased the risk of NEC [39]. In the current study, ibuprofen was also used to treat patent ductus arteriosus in 8% of the total studied infants, but without differences among groups. Likewise, some causes of ischemic intestinal necrosis, such as a duration of oxygen saturations < 85%, were similar among groups, while major congenital heart disease was an exclusion criterion in the current study [40]. Other known risk factors for NEC were also similar among groups [2,6], and no patients had hemoglobin ≤ 8 g/dL [41]. 

The percentage of alpha-linolenic acid was higher in erythrocytes from the DHA-group compared with those in the infants from the control-group and NEC-group (Table 5). However, this difference is probably of little relevance because alpha-linolenic acid has low anti-inflammatory potential compared with DHA [42,43].

The current study has several strengths. The trial was blinded, randomized, and placebo controlled. NEC was the primary outcome and was confirmed. The trial was amply powered for this outcome. The DHA and control interventions were delivered by clinical staff and administration was supervised by research staff to ensure compliance. Loss to follow-up was limited. Both intent-to-treat and per protocol analysis were consistent. The DHA dosage was regularly adjusted to maintain similar daily DHA delivery per kg of body weight. The two groups had similar characteristics and covariates, possibly due to the randomization in the blocks of birth weight and recruiting center. Unfortunately, nearly 85% of mothers were not able to provide their milk for feeding infants, but this allowed us to evaluate the DHA effect without the important protective effect of human milk. 

There are also some limitations. We did not assess the fatty acid status at the end of the intervention. Moreover, we did not assess markers of immune function or inflammation or fecal microbiota. Additionally, these results are not generalizable to extremely low birth weight infants because the lower limit of birthweight used for patient recruitment permitted by the Mexican National Committee of Scientific Research was 1 kg. Therefore, whether this intervention is useful to prevent NEC development in preterm infants with a lower weight and gestational age at birth needs to be elucidated.

## 5. Conclusions

A daily enteral dose of DHA for 14 days starting with the first enteral feeding may be a preventive strategy for NEC in preterm infants.

## Figures and Tables

**Table 1:** Fatty acid composition of the intervention (docosahexaenoic acid (DHA)-rich) and sham oils (%wt of total fatty acids).

Fatty Acid	GROUP	
	DHA Intervention (Algal Oil)	Control (High-Oleic Sunflower Oil)
Capric	1.0	0.0
Lauric	4.3	0.0
Myristic	14.0	0.1
Palmitic	12.2	5.2
Palmitoleic	1.9	0.1
Stearic	0.7	3.8
Oleic	18.9	58.9
Linoleic	1.3	29.2
Gamma-Linolenic	0.4	0.3
Alpha-Linolenic	<0.1	1.2
Eicosenoic	<0.1	0.3
Arachidonic	<0.1	0.0
Eicosapentaenoic	<0.1	0.0
Behenic	0.2	0.7
Docosahexaenoic	44.3	0.0
Nervonic	0.1	0.0

These fatty acids represent more than 99% of the total fatty acid profile.

**Table 2:** Characteristics of the preterm infants.

	GROUP		*p*
	DHA *n* = 100	Control *n* = 100	
**At Birth**			
Born by cesarean section, *n* (%)	96 (96)	99 (99)	0.391
Received antenatal steroid, *n* (%)	48 (48)	42 (42)	0.601
Gestational age, weeks	30 (29, 32)	31 (30, 32)	0.513
Male sex, *n* (%)	52 (52)	47 (47)	0.572
Apgar score at minute 5 ≥ 7, *n* (%)	96 (96)	95 (95)	1.000
**At Baseline**			
Corrected gestational age, weeks	31.0 (29.8, 32.3)	31.1 (28.0, 32.5)	0.306

Data are presented as the median (Q25, Q75) unless otherwise stated.

**Table 3:** Clinical course and medical management during the hospital stay of the very low birth weight infants stratified by confirmed necrotizing enterocolitis (NEC).

	GROUP			*p*
	DHA *n* = 100	Control *n* = 93	NEC *n* = 7	
Birthweight 1000–1250 g, *n* (%)	40 (40)	44 (47)	4 (57)	0.460
Birthweight 1251–1500 g, *n* (%)	60 (60)	49 (53)	3 (43)	
Small for gestational age, *n* (%)	14 (14)	12 (13)	1 (14)	0.974
Twins, *n* (*%*)	23 (23)	30 (32)	2 (29)	0.354
Severe asphyxia at birth, *n* (*%*)	9 (9)	10 (11)	0	0.627
Severity of disease (CRIB) at baseline	2.0 (1, 5.0)	1.0 (1, 4.5)	1.0 (0, 6.0)	0.760
Hemoglobin at baseline, g/dl	15.7 (14.2, 17.7)	16.5 (14.8, 18.0)	14.1 (12.9, 16.8)	0.150
Respiratory distress syndrome, *n* (*%*)	90 (90)	84 (90)	7 (100)	0.681
Suspected or confirmed sepsis, *n* (*%*)	66 (66)	57 (61)	7 (100)	0.112
PDA, *n* (*%*)	18 (18)	21 (23)	3 (43)	0.259
Apnea, *n* (*%*)	36 (36)	24 (26)	4 (57)	0.110
Apnea events in week 1, *n*	1 (1, 2)	1 (1, 2)	1	0.780
Apnea events in week 2, *n*	2 (1, 2)	1.5 (1, 3)	1 (1, 1.5)	0.669
Apnea events during hospitalization, *n*	3.0 (2, 5)	3.0 (1, 7)	4.5 (1.3, 8)	0.881
Requirement for phase III ventilator support, *n* (*%*)	65 (66)	62 (67)	6 (86)	0.552
Duration with SpO_2_ < 85% in week 1, h	5.5 (2.0, 12.0)	4 (2.0, 6.0)	0	0.219
Duration with SpO_2_ < 85% in week 2, h	7.0 (5.0, 13.5)	4 (3.5, 8.0)	0	0.146
Retinopathy of the prematurity, *n* (*%*)	23 (23)	23 (25)	0*	0.325
Antibiotics used, *n* (*%*)	97 (97)	92 (99)	7 (100)	0.589
Postnatal age at antibiotic start, h	8 (4, 24)	8 (4, 24)	8 (4, 48)	0.937
Antibiotic duration, days	26 (17, 42)	27 (19, 42)	28 (25, 38)	0.950
Postnatal steroid use, *n* (*%*)	21 (21)	21 (23)	3 (43)	0.408
Ibuprofen use, *n* (*%*)	9 (9)	6 (7)	1 (14)	0.665
Omeprazole during intervention period, *n* (*%*)	43 (43)	41 (46)	2 (29)	0.671
Duration of omeprazole use during intervention period, days	4 (1, 6)	4 (2, 7)	4.5 (3, 6)	0.759
Infants with RBC transfusion during intervention period, *n* (*%*)	38 (38)	32 (34)	3 (43)	0.821
Number of RBC transfusions during intervention period, *n*	1 (1, 1)	1 (1, 1)	1.5 (1, 2)	0.467
Length of NICU stay, days	9.5 (2, 18)	12.0 (3, 21)	18.0 (9, 31)	0.166
Length of hospital stay, days	45 (35, 55)	46 (38, 53)	47 (26, 66) *	0.888

Data are presented as the median (Q25, Q75) unless otherwise stated. All infants were from the control-group; CRIB, clinical risk index for babies with weight at birth < 1500 g; PDA, persistence of ductus arteriosus; SpO_2_, oxygen saturation measured with a pulse oximeter; RBC, red blood cell; NICU, neonatal intensive care unit. * Not determined in three infants due to death.

**Table 4:** Nutritional support of the preterm infants during the hospital stay.

Nutritional intake variable	GROUP			*p*
	DHA *n* = 100	Control *n* = 93	NEC *n* = 7	
**Week One of Postnatal Age**				
Total fluid volume, mL/kg/day	100 (88, 108)	97 (88, 105)	89 (75, 104)	0.437
Infants receiving TPN before EF, *n* (%)	50 (57)	48 (59)	6 (86)	0.327
Postnatal age at starting TPN, days	2.0 (1.7, 2.4)	2.7 (1.0, 2.4)	3.0 (1.8, 4.0)	0.851
Lipid supply by TPN, g/kg/day	2.0 (0.6, 3.8)	2.1 (0.4, 2.9)	2.0 (1.4, 2.4)	0.877
Postnatal age at starting EF, days	4.0 (2.0, 6.5)	4.3 (2.5, 6.2)	7.0 (4.1, 9.1)	0.176
Lipid intake-EFF, g/kg/day	1.1 (0.5, 2.3)	1.2 (0.6, 2.0)	0.5 (0.2, 1.2)	0.151
Fatty Acid Intake from EFF, mg/kg/day				
Linoleic acid	69 (32, 231)	100 (31, 217)	38 (11, 107)	0.282
Alpha-linolenic acid	26 (10, 44)	23 (10, 39)	9 (4, 22)	0.154
Arachidonic acid	5 (2, 9)	5 (2, 8)	2 (0.8, 5)	0.171
Docosahexaenoic acid	5 (2, 9)	5 (2, 8)	2 (0.8, 5)	0.161
**Week Two of Postnatal Age**				
Total fluid volume, mL/kg/day	136 (125, 147)	138 (123, 147)	127 (99, 131)	0.081
Lipid supply by TPN, g/kg/day	1.8 (0.8, 2.4)	1.8 (0.8, 2.3)	2.1 (1.4, 2.5)	0.717
Lipid intake-EFF, g/kg/day	4.5 (2.5, 5.8)	4.2 (2.9, 5.7)	1.2 (0.4, 3.9)	0.059
Fatty Acid Intake from EFF, mg/kg/day				
Linoleic acid	576 (209, 880)	506 (119, 833)	82 (21, 505)	0.106
Alpha-linolenic acid	86 (48, 111)	79 (35, 108)	23 (8, 74)	0.064
Arachidonic acid	17 (10, 22)	16 (7, 22)	7 (1.4, 15)	0.088
Docosahexaenoic acid	17 (10, 22)	16 (7, 22)	7 (1.4, 15)	0.088
**Human Milk Intake**				
Infants fed any volume of human milk, *n* (%)	18 (18)	13 (14)	0	0.382
Infants fed human milk during first week post-enteral feeding, *n* (%)	11 (11)	4 (4.2)	0	0.157
Intake during first week post-enteral feeding, mL/kg/day	3.9 (2.1, 10.3) ^a^	4.9 (0.7, 9.9) ^b^	0 ^c^	0.003
Infants fed human milk during second week post-enteral feeding, *n* (%)	11 (11)	6 (6.5)	0	0.376
Intake during second week post-enteral feeding, mL/kg/day	8.3 (2.4, 17.2) ^a^	5.4 (1.8, 20.3) ^b^	0 ^c^	0.052
Infants fed human milk during third week post-EF, *n* (%)	9 (9)	7 (7.5)	0	0.679
Intake during third week post-enteral feeding, mL/kg/day	6.7 (2.6, 23.1) ^a^	7.9 (4.1, 13.6) ^a^	0 ^b^	0.029
Infants fed human milk during fourth week post-EF, mL/kg/day, *n* (%)	12 (12)	7 (7.5)	0	0.390
Intake during fourth week post-enteral feeding, mL/kg/day	5.6 (2.6, 12.7) ^a^	4.3 (1.5, 6.2) ^b^	0 ^c^	0.043
Intake during hospital stay, mL/kg/day	14 (7, 37)	12 (3, 60)	0	0.448
Time required to reach full enteral feeding, days	15 (12, 22)	17 (12, 22)	20 (16, 46)	0.161

Data are presented as median (Q25, Q75) unless otherwise stated. All infants were from the control group. Different superscripts indicate significant differences among groups (*p* < 0.05, Mann–Whitney-U test). TPN, total parenteral nutrition; EF, enteral feeding; and EEF, enteral feeding from formula. Note: The calculated intake of DHA is from formula in both groups and excludes the additional supplemental intake of 75 mg/kg/day in the DHA-group.

**Table 5:** Fatty acid profile of erythrocyte membranes from preterm infants and of human milk (%weight of total fatty acids).

Fatty Acid	DHA *n* = 100	Control *n* = 93	NEC *n* = 7	*p*	DHA *n* = 100	Control *n* = 93	NEC *n* = 7
In Baseline Erythrocyte Membranes					In Human Milk during Hospital Stay		
Lauric	0.34 (0.15, 0.67)	0.38 (0.22, 0.79)	0.36 (0.15, 0.67)	0.534	7.6 (7.0, 9.6)	6.6 (6.6, 7.2)	- *
Myristic	0.71 (0.57, 1.06)	0.80 (0.61, 1.24)	1.02 (0.71, 1.06)	0.252	9.0 (7.7, 9.5)	8.8 (8.2, 9.4)	- *
Palmitic	32.5 (29.9, 40.7)	33.4 (32.2, 42.3)	31.7 (30.3, 48.0)	0.463	22.0 (20.1, 26.9)	24.7 (24.2, 25.2)	- *
Palmitoleic	1.12 (0.78, 1.42)	1.18 (0.80, 1.48)	1.47 (1.0, 2.50)	0.055	2.3 (2.1, 2.6)	2.3 (2.2, 2.4)	- *
Stearic	17.4 (15.9, 18.9)	16.9 (4.3, 23.6)	17.1 (15.8, 19.0)	0.485	6.6 (6.1, 6.7)	7.0 (6.6, 7.3)	- *
Oleic	16.7 (15.2, 18.8)	16.6 (14.6, 19.3)	16.7 (16.0, 18.8)	0.894	33.7 (31.6, 34.9)	32.0 (31.9, 32.9)	- *
Linoleic	5.1 (4.0, 7.4)	5.0 (4.1, 7.3)	3.6 (2.2, 7.3)	0.473	16.1 (15.9, 17.4)	15.5 (14.4, 17.0)	- *
Alpha-linolenic	0.12 ^a^ (0.09, 0.30)	0.10 ^b^ (0.07, 0.17)	0.07 ^b^ (0.07, 0.17)	0.042	1.2 (1.1, 1.5)	1.1 (1.0, 1.2)	- *
Arachidonic	14.67 (5.07, 20.11)	15.50 (5.51, 20.2)	13.5 (4.3, 18.9)	0.829	0.6 (0.5, 0.6)	0.5 (0.5, 0.6)	- *
Eicosapentaenoic	0.54 (0.24, 0.83)	0.53 (0.27, 0.70)	0.40 (0.31, 0.69)	0.782	0.03 ^a^ (0.03, 0.06)	0.10 ^b^ (0.09, 0.12)	- *
Nervonic	2.82 (2.0, 3.4)	2.62 (2.11, 3.4)	2.90 (2.0, 3.1)	0.896	0.03 (0.04, 0.05)	0.03 (0.03, 0.04)	- *
Docosahexaenoic	2.92 (1.2, 3.4)	2.99 (0.95, 3.7)	3.0 (2.0, 4.0)	0.812	0.22 ^a^ (0.16, 0.27)	0.35 ^b^ (0.33, 0.36)	- *

Data are presented as median (Q25, Q75). * No infant who developed NEC received human milk. Different superscripts indicate significant differences between groups (*p* < 0.05; Mann–Whitney-U test).

## Data Availability

Data can be made available by contacting the corresponding author.

## Clinical Trial registration ID

NCT01745510. https://clinicaltrials.gov/ct2/show/NCT01745510.
